# Supplementary material for: Therapy-Induced Senescent/Polyploid Cancer Cells Undergo Atypical Divisions Associated with Altered Expression of Meiosis, Spermatogenesis and EMT Genes
Source: Int J Mol Sci. 2022 Jul 27;23(15):8288. doi: 10.3390/ijms23158288 (PMC9368617; doi:10.3390/ijms23158288)
Supplement: Supplementary file 1 [file ijms-23-08288-s001.zip › ijms-1764668-supplementary.pdf]

Supplementary Materials:

(a)

HCT116 MCF-7 MDA-MB-231

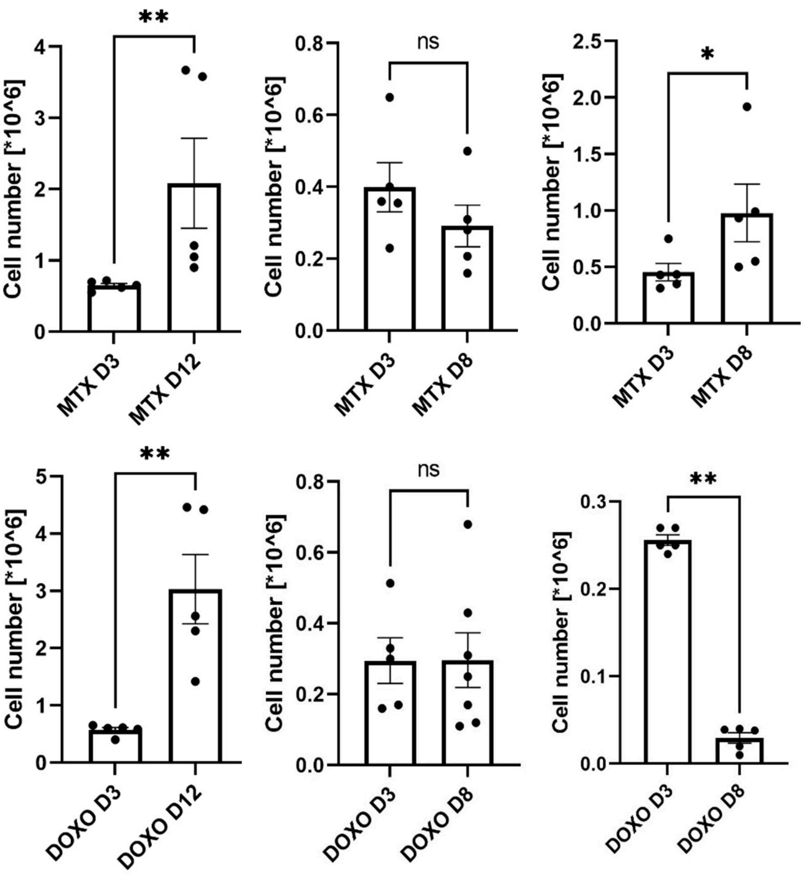

Suppl. Figure S1. Proliferation of various cancer cells is temporarily arrested after different chemotherapeutics (a) HCT116 colon cancer cells, MCF-7 and MDA-MB_231 breast cancer cells counted on day 3 and day 8 or 12 after 24 hour treatment with the indicated drug.

Bars represent mean of at least 3 independent experiments, error bars: SEM. Statistical significance: 0.01 < P < 0.05, * 0.05 < P <0.001, ***P < 0.001.

(a)

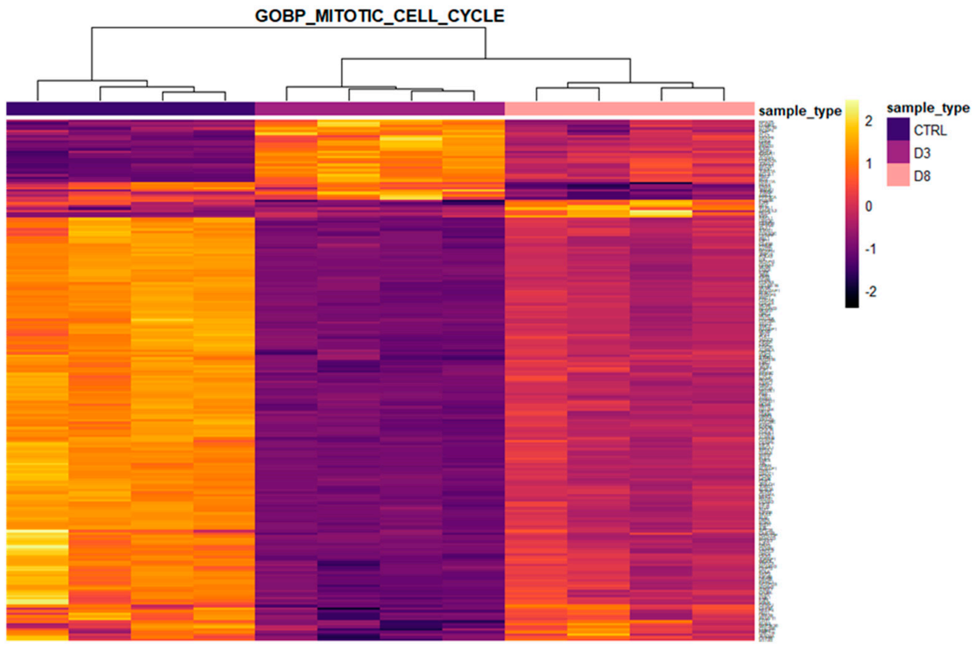

(b)

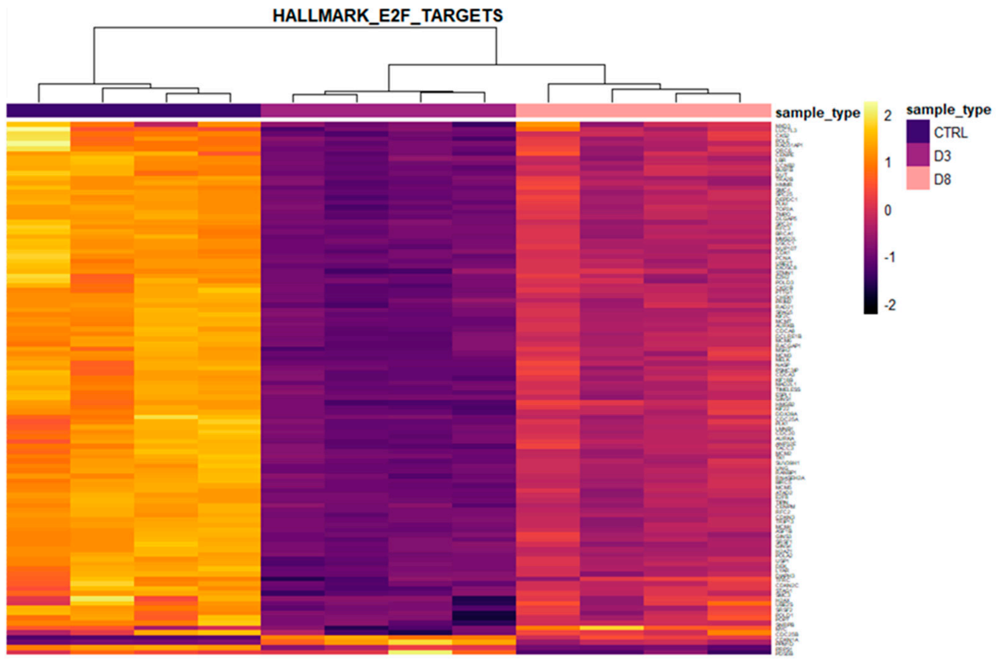

Suppl. Figure S2. Rescue of proliferation related genes during Escape from Senescence (a) Heatmap representing GOBP Mitotic Cell Cycle prepared on Differentially Expressed genes from the geneset. **b)** Heatmap representing HALLMARK E2F Targets prepared on Differentially Expressed genes from the geneset.
